# Biodegradable magnesium based metal materials inhibit the growth of cervical cancer cells

**DOI:** 10.1038/s41598-024-63174-w

**Published:** 2024-09-02

**Authors:** Xiaojing Nie, Lei Wang, Zexiang Zhao, Jingxin Yang, Chen Lin

**Affiliations:** 1 Department of Pathology, School of Basic Medical Sciences, Xinjiang Medical University, Ürümqi, 830017 Xinjiang People’s Republic of China; 2 School of Public Health, Xinjiang Medical University, Ürümqi, 830017 Xinjiang People’s Republic of China; 3 Beijing Engineering Research Center of Smart Mechanical Innovation Design Service, Beijing Union University, No.4 Gongti North Road, Chaoyang District, Beijing, 100027 People’s Republic of China; 4 Institute of Medical Sciences, Xinjiang Medical University, Xinjiang, China

**Keywords:** Magnesium, Biomaterials, Cervical cancer, Degradation, Tumor, Biological techniques, Medical research, Materials science

## Abstract

Traditional chemotherapy drugs for cervical cancer often cause significant toxic side effects and drug resistance problems, highlighting the urgent need for more innovative and effective treatment strategies. Magnesium alloy is known to be degradable and biocompatible. The release of degradation products Mg^2+^, OH^−^, and H_2_ from magnesium alloy can alter the tumor microenvironment, providing potential anti-tumor properties. We explored the innovative use of magnesium alloy biomaterials in the treatment of cervical cancer, investigating how various concentrations of Mg^2+^ on the proliferation and cell death of cervical cancer cells. The results revealed that varying concentrations of Mg^2+^ significantly inhibited cervical cancer by arresting the cell cycle in the G0/G1 phase and inducing apoptosis in SiHa cells, effectively reducing tumor cell proliferation. In vivo experiments demonstrated that 20 mM Mg^2+^ group had the smallest tumor volume, exhibiting a potent inhibitory effect on the biological characteristics of cervical cancer. This enhances the therapeutic potential of this biomaterial as a local anti-tumor therapy and lays a theoretical foundation for the potential application of magnesium in the treatment of cervical cancer.

## Introduction

Cervical cancer represents a prevalent cause of mortality among women. In its early stages, it often presents no significant clinical symptoms. Cervical cancer typically presents no noticeable symptoms in its early stages. In advanced stages, the life of the patient is severely endangered as the tumor invades nearby tissues and organs^1,2^. Currently, patients with early-stage cervical cancer often undergo radical surgery, while those with larger tumors, inflammation, or metastasis are treated with radiation or chemotherapy^3^. Cisplatin and paclitaxel liposomes are frequently used chemotherapeutic agents for cervical cancer in a clinic but with the drug resistance caused by their widespread use, as well as many toxic and side effects such as anaphylaxis, myelosuppression, ototoxicity, nephrotoxicity, and gastrointestinal reactions^4,5^. Surgical resection followed by radiotherapy or chemotherapy is a commonly used treatment approach in clinical practice^6^. However, due to poor dose control of radiotherapy and chemotherapy, platform based on biomaterials are often used to achieve slow release of chemotherapy drugs and control systemic toxicity^7^. Therefore, enhancing drug targeting and mitigating tumor multidrug resistance and side effects remain significant challenges in antitumor drug research.

It has been reported that magnesium (Mg) and its alloys may exert potential anti-tumor activity through the following ways: the release of degradation products Mg^2+^, hydroxide ion (OH^-^) and hydrogen (H_2_) can inhibit tumor proliferation by accumulating of free radicals^8^. The release of Mg^2+^ can inhibit tumor cells in the G0/G1 phase of the cell cycle. The extracellular pH of tumor cells is typically weakly acidic, ranging from 5.7 to 7.0^9^. The release of OH^−^ increases the extracellular pH of tumor cells, which is not conducive to the growth of tumor cells, Mg activates over 300 enzymes via interaction with the TRPM7 plasma channel α-kinase. This interaction leads to the phosphorylation of downstream substrates involved in cell differentiation, proliferation, migration, and apoptosis. Due to its biocompatibility and degradability, Mg based materials can be developed as active agents in drug release systems and corresponding coatings. In addition, the degradation of Mg based materials triggers surface proximity effects, such as an increase in pH value, osmotic pressure, and hydrogen evolution. These effects can be controlled by adjusting the degradation rate of the materials. These surface proximity effects also have the potential to directly target cancer cells and reduce tumor growth. Recent reports indicate a dual role for Mg^2+^, while Mg^2+^ supplementation following deficiency can increase tumor growth in mice, Mg^2+^ deficiency itself is linked to an enhanced pro-inflammatory response, as well as the activation and metastasis of vascular endothelial cells. This suggests that Mg^2+^ has a complex role in tumor development, promoting growth in the early stages and inhibiting it in advanced solid cancers. The effectiveness of magnesium-based materials in this context is uncertain^10,11^.

Consequently, we investigated how different concentrations of Mg^2+^ affect the proliferation and cell death of cervical cancer cells. Observations unveiled that Mg^2+^ effectively inhibits the proliferation of cervical cancer cells and promotes their apoptosis. Mg^2+^ hinders the progression of the cell cycle within the G0/G1 phase and diminishes the presence of ROS. In vitro experiments indicate that the degradation of Mg significantly impedes tumor growth compared to the control group. To the best of our understanding, this is the first documentation of Mg degradation’s inhibitory effects on cervical cancer. Our exploration lays down a theoretical groundwork for potential employment of Mg-based metal materials in the treatment of cervical cancer.

## Results

### Cell proliferation test

**Figure 1:**
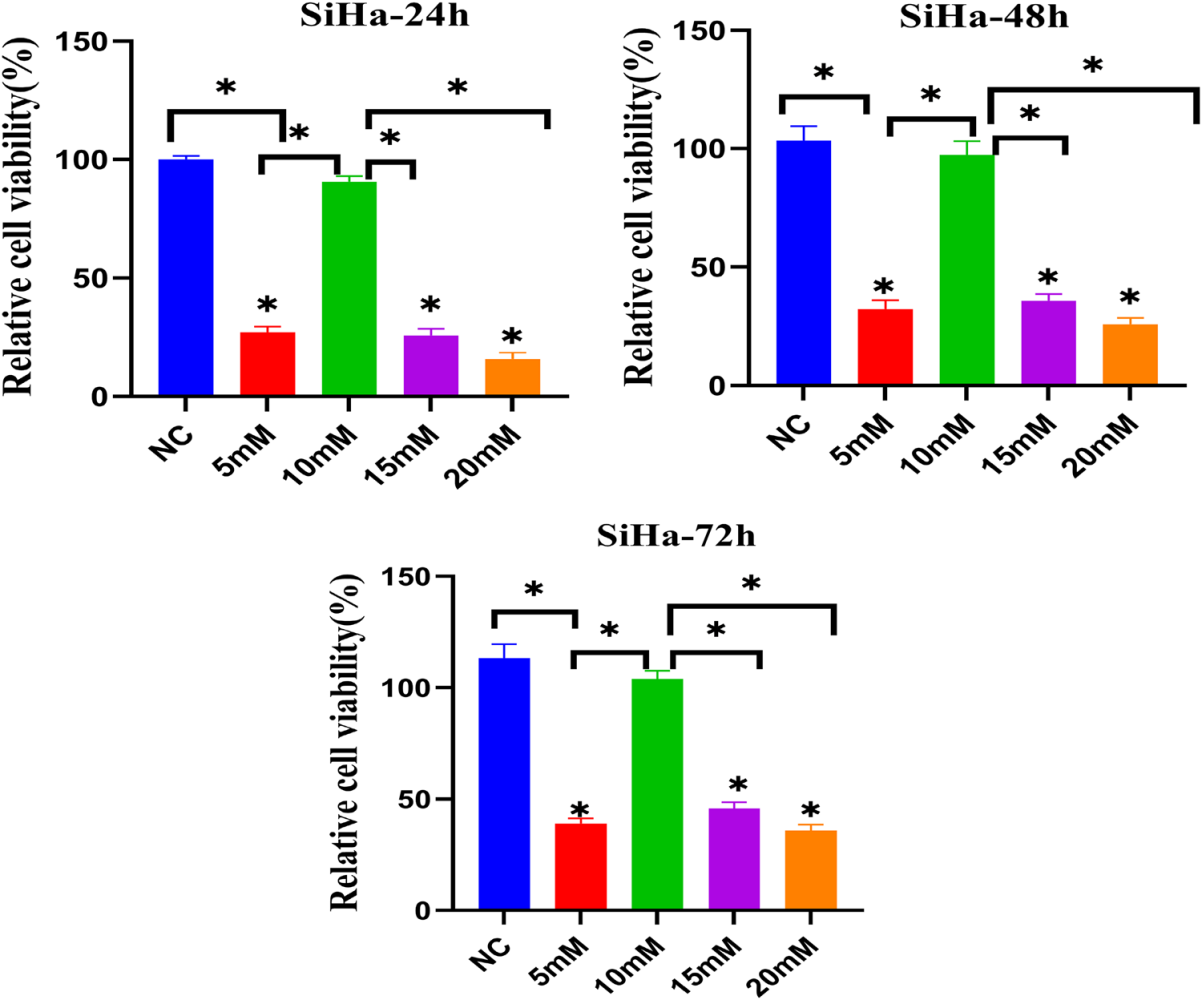
OD values of NC (control group), 5 mM Mg^2+^ group, 10 mM Mg^2+^ group, 15 mM Mg^2+^ group, and 20 mM Mg^2+^ group after incubation of SiHa cells for 24 h, 48 h, and 72 h. One-way ANOVA (n = 3 per treatment group). *p < 0.05 versus control group.

SiHa cells were cultured under four distinct conditions, including NC (control group), 5 mM, 10 mM, 15 mM, and 20 mM Mg^2+^ groups and divided into 4 groups which cells were cultured by 24 h, 48 h and 72 h. Cells were compared for cell proliferation by CCK-8 assay. We found that compared to the control group in 24 h, 48 h, and 72 h, SiHa cells proliferation viability is relatively low in the 5 mM, 15 mM, and 20 mM Mg^2+^ groups, with statistical significance. Moreover, compared between groups, 20 mM Mg^2+^ group had the lowest cell proliferation and had significant statistical significance. The results demonstrated that the 20 mM Mg^2+^ group, the stronger the inhibition of SiHa cells proliferation (Fig. 1).

### Staining of live/dead cell detection

**Figure 2:**
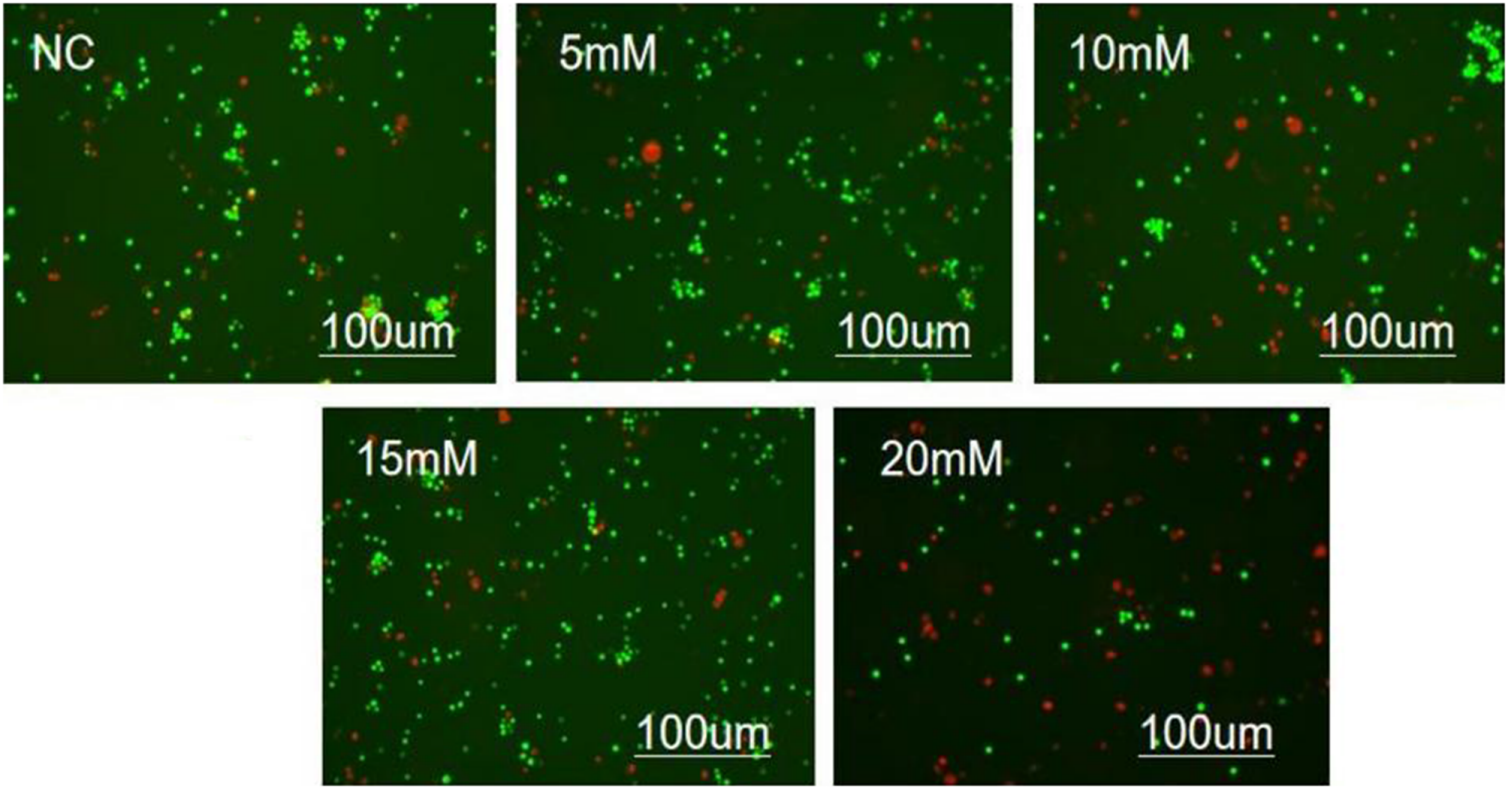
Observation of Live/dead cells in each group cultured with SiHa cells for 24 h using fluorescence microscope (Red is dead cells, green is live cells).

The assessment of cell viability was carried out using the LIVE/DEAD staining assay, as demonstrated in Fig. 2. After a 48-h exposure to different concentrations, including the control group (NC), 5 mM, 10 mM, 15 mM, and 20 mM Mg^2+^ groups, the number of dead cells (indicated by the red color) was examined across these five groups. The results demonstrate a clear correlation between the increasing concentration of Mg^2+^ and the escalating number of deceased cells.

### ROS detection

**Figure 3:**
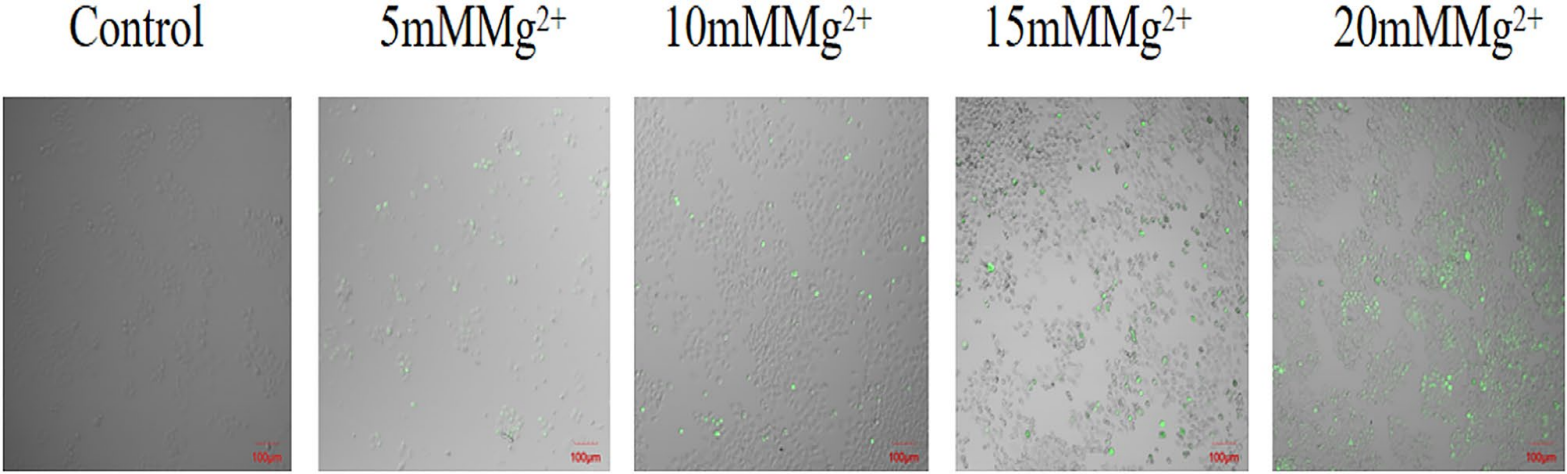
The effect of different concentrations of Mg^2+^on ROS in SiHa cells (scale bar: 100 μm).

The findings depicted in Fig. 3 exhibit a pronounced disparity in the magnitude of reactive oxygen species (ROS) among the NC group, 5 mM, 10 mM, 15 mM, and 20 mM Mg^2+^ groups. Microscopic analysis reveals a consistent trend wherein an increase in Mg^2+^ concentration correlates with increase in ROS levels, signifying a clear inhibitory impact on SiHa cells. This study indicates that the increase of Mg^2+^ promotes the level of ROS and inhibits the growth of tumor cells.

### Cell cycle analysis

**Figure 4:**
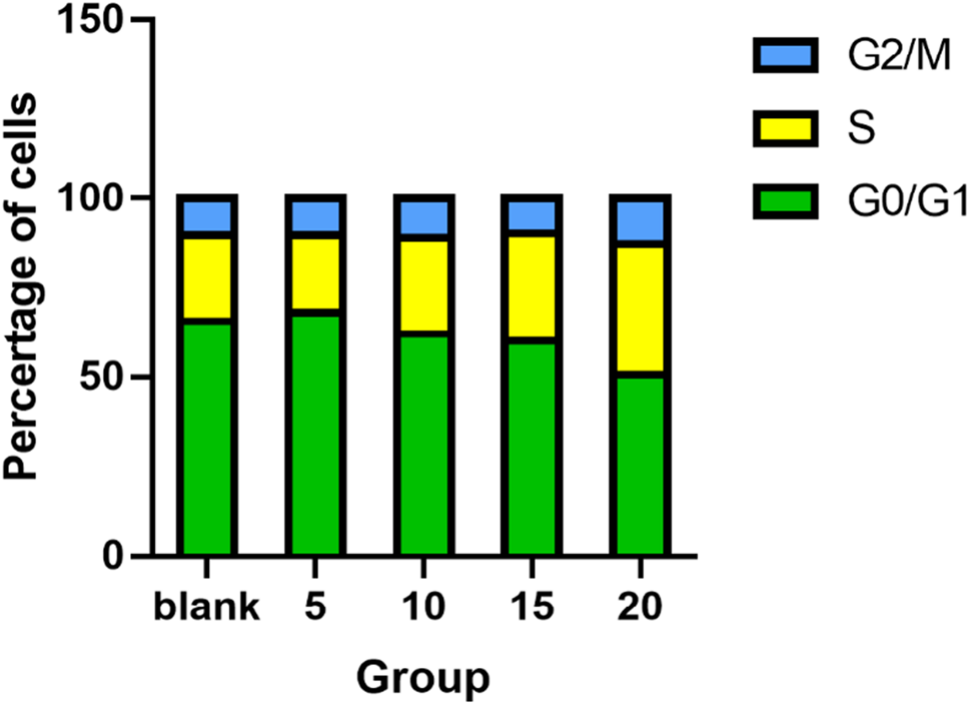
The effect of different Mg^2+^ on the cell cycle of SiHa cells.

SiHa cells were subjected to four distinct culture conditions, including the 5 mM Mg^2+^ group, 10 mM Mg^2+^ group, 15 mM Mg^2+^ group, and 20 mM Mg^2+^ group, with each group undergoing a 48-h culturing period. The findings indicate that the 20 mM Mg^2+^ concentration induces cell cycle arrest specifically in the G0/G1 phase. This outcome manifests as a conspicuous inhibitory effect on SiHa cells (Fig. 4).

### Cell apoptosis analysis

**Figure 5:**
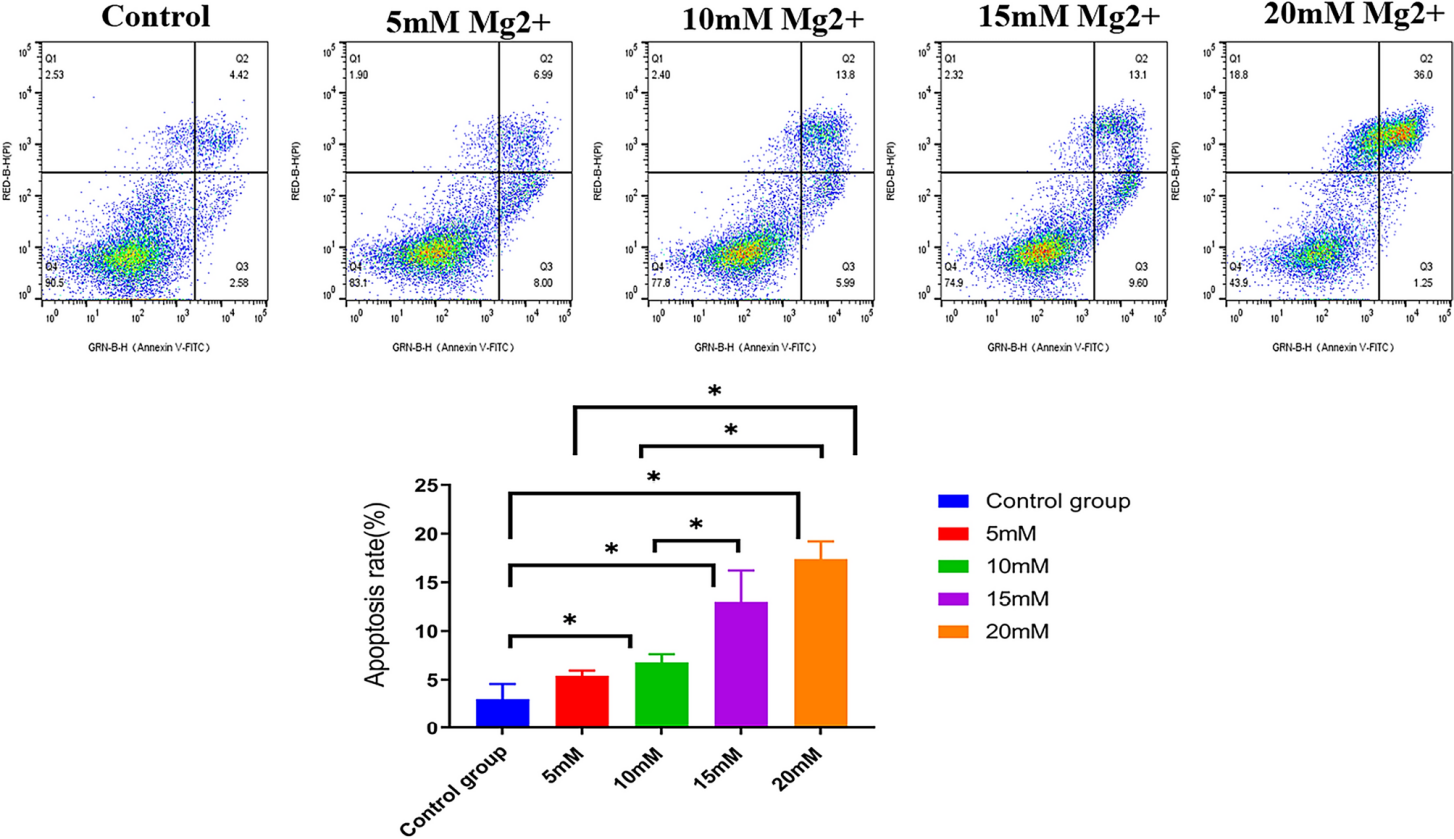
The results showed that among the control group, 5 mM Mg^2+^ group, 10 mM Mg^2+^ group, 15 mM Mg^2+^ group, and 20 mM Mg^2+^ group. *p < 0.05.

SiHa cells were subjected to four different culture conditions consisting of the 5 mM, 10 mM, 15 mM, and 20 mM Mg^2+^ groups, with each group cultured for a period of 48 h. The results demonstrate that the 20 mM Mg^2+^ concentration triggers cell cycle arrest exclusively in the G0/G1 phase. This outcome is observed as a significant inhibitory effect on SiHa cells (Fig. 5).

### Western blotting

**Figure 6:**
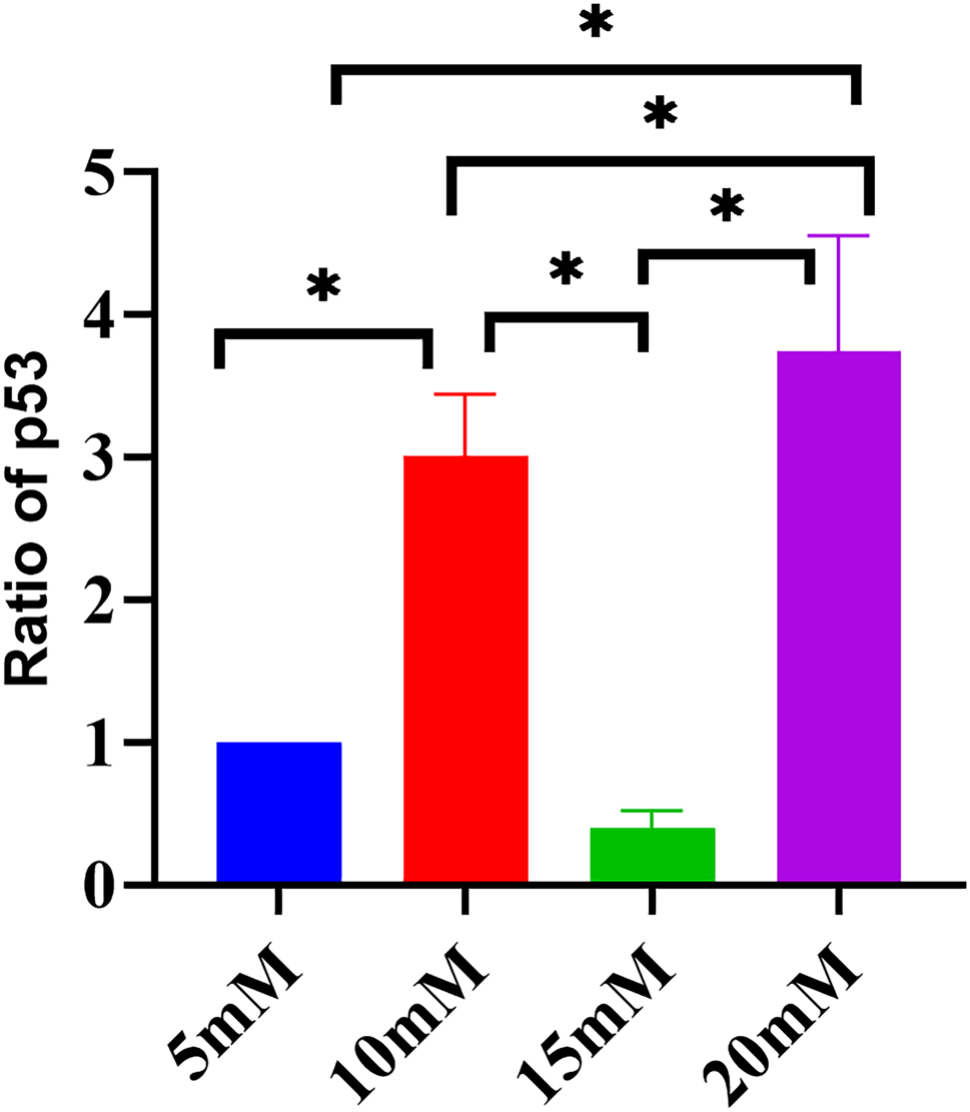
After 7 days of incubation, the 5 mM (5 mM Mg^2+^group), 10 mM (10 mM Mg^2+^ group), 15 mM (15 mM Mg^2+^ group), 20 mM (20 mMMg^2+^ group) that protein levels p53 of expression one-way ANOVA (n = 3 per treatment group). 20 mM Mg^2+^ group has highest the level of p53 protein. *p < 0.05. The samples derive from the same experiment and that blots were processed in parallel and the original blots are shown in Supplementary Fig. 1.

The different groups of protein levels p53 were tested by western blotting. The data from Western blotting indicated that compared with the 5 mM, 10 mM, 15 mM, 20 mM Mg^2+^ groups of p53 protein levels expression had increased obviously. There were significant differences between all groups, especially the 20 mM Mg^2+^ group had arrived at the highest protein levels of expression. Figure 6 results showed that the higher the concentration of Mg^2+^, the higher the level of p53, which had an obvious inhibitory effect on SiHa cells.

### In vivo experiments

**Figure 7:**
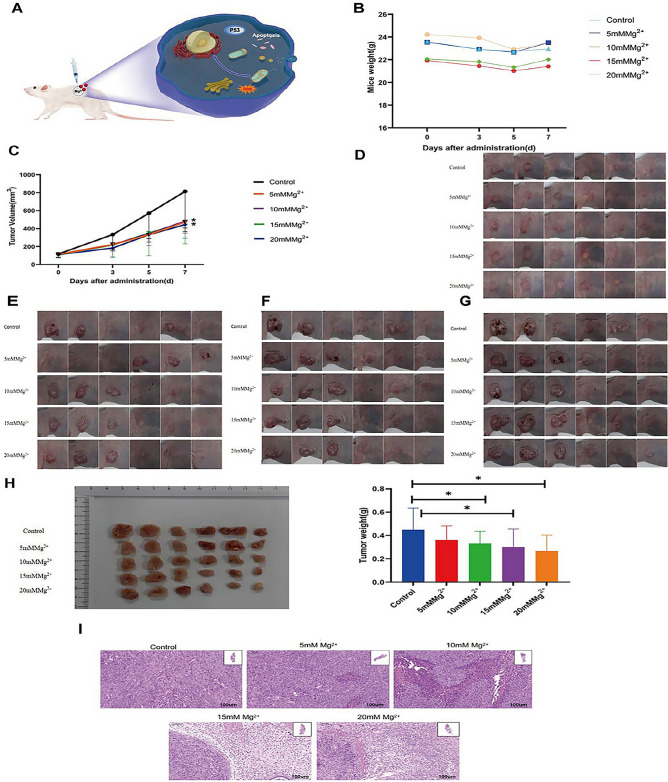
(**A**) Subcutaneous injection of different concentrations of Mg^2+^ for anti-tumor treatment and its mechanism. (**B**)The results of average weight changes in different time points and groups showed that there was no significant change in the weight of mice in each group. (**C**) The average tumor volume changes in different time points and groups. The results showed that compared with the Control group, the tumor volume of the 15mMMg^2+^and 20mMMg^2+^groups was smaller, with a significant difference (*p* < 0.05). (**D**–**G**) Tumor growth status of each group on 0, 3, 5, 7 day (M01, M02, M03, F01, F02, F03 in each group). (**H**) After 7 days, the mice were euthanized and the tumor was removed from the mice. Remove the mouse tumor and measure its volume, weight, and take photos of its morphology. (**I**) After 7 days, the mice were euthanized and the tumor was removed from the mice. Remove the mouse tumor and fix with formalin, embed in paraffin, and stain with H & E (scale bar: 100 μm).

As shown in Fig. 7H, compared with the blank control group, there was a significant statistical difference between 15 mMMg^2+^ group and 20 mMMg^2+^group. Especially the 20 mMMg^2+^group with the smallest tumor volume (*p < 0.05).

## Discussion

In recent years, Mg and its alloys have gained recognition for their biodegradability and excellent biocompatibility^12^. Biodegradable Mg alloys, particularly in the context of orthopedic implants, have garnered increasing attention. Additionally, several studies have highlighted the potential antitumor activity of Mg, highlighting its crucial role in supporting intracellular processes against tumor development. Intracellular Mg plays a pivotal role in regulating enzymatic reactions that are essential for maintaining DNA stability, including mismatch repair, base excision, and nucleotide excision^13,14^. Due to its relatively low standard reduction potential (− 2.37 VSHE), Mg has the capacity to spontaneously degrade into Mg hydroxide and emit hydrogen when exposed to physiological or buffered environments^15^. The regulated degradation of Mg biomaterials has been established as safe and well-tolerated in both animal studies and human clinical trails^16,17^. Its degradation in vivo can create a gas environment rich in H_2_ up to 1.46 mM near the implant^18^, which exceeds the saturation concentration of hydrogen in water (0.8 mM). The use of Mg implants offers significant advantages over traditional H_2_ inhalation therapy, including accelerated H_2_ release in the tumor microenvironment (TME) to increase local hydrogen concentration, ease of transplantation without the need for direct H_2_ inhalation, and the capacity of 1 cm^3^ of pure Mg implants to generate up to 1600 mL of hydrogen, enhancing energy production around the tumor^19^. Therefore, we believe that the degradable Mg implant has a better effect in transferring H_2_ to tumor tissue than inhaling H_2_. The degradation of Mg is accompanied by the constant release of ions and H_2_, as well as the increase of pH value and osmotic pressure in the microenvironment of the material surface. Currently, there is no literature that reports on how these degradation products will play an anti-tumor role on cervical cancer cells. However, the primary challenge to the clinical application of Mg base metals is their rapid and uncontrollable corrosion or degradation when exposed to chlorides, such as those found in body fluids^20–23^. Therefore, a major challenge in using Mg-based materials for cancer treatment is conducting in-depth studies on their specific cytotoxic effects on cervical cancer cells and assessing the integrity of adjacent healthy cells. Research reports that Mg and its alloys may exert potential anti-tumor activity through the following ways: the release of degradation products Mg^2+^, OH^−^ and H_2_ can inhibit tumor proliferation through the accumulation of free radicals. The release of Mg^2+^ and H_2_ can inhibit tumor cells in G0/G1 phase of the cell cycle. Additionally, the release of OH^-^ increases the pH value outside the tumor cells and is not conducive to the growth of tumor cells^24^. Wang et al. ^25^ showed that high concentration (18 mmol/L) of Mg^2+^ has toxic effect on cells. Furthermore, the inquiry into the mechanism underlying the anti-SiHa cell effects of Mg was extended to in vitro investigations. Research study: SiHa cells were cultured with various Mg^2+^ concentrations (control, 5 mM, 10 mM, 15 mM, 20 mM), and the cell inhibition rate was measured at 24 h, 48 h, and 72 h. Figure 1 results demonstrated that the 20 mM Mg^2+^ group exhibited the strongest the inhibition of SiHa cells proliferation, with the highest cell inhibition rate being statistical significance. At various time points, no significant difference was observed in cell viability between the 10 mM Mg^2+^ group and the control group, indicating that the inhibitory effect of the 10 mM Mg^2+^ group on tumor cells was relatively poor and different concentrations of Mg^2+^ had different effects on tumor cells. These findings are consistent with previous research. ROS test results (Fig. 3) demonstrated a significant difference in ROS level among NC (control group), 5 mM, 10 mM, 15 mM, and 20 mM Mg^2+^ groups. Microscopic observations revealed that higher Mg^2+^ concentration led to increased ROS levels, significantly inhibiting SiHa cell proliferation. Apoptosis, a key clinical marker for anticancer therapy, was assessed across different Mg^2+^ concentrations^26,27^. Figure 5 results demonstrated that there was a notable difference in Apoptosis level among the control group, 5 mM, 10 mM, 15 mM, and 20 mM Mg^2+^ groups. The results showed that higher Mg^2+^ concentrations resulted in increased apoptosis rates, significantly inhibiting SiHa cell growth. The higher the level of Apoptosis rate, which had an obvious inhibitory effect on SiHa cells. Based on cell viability and apoptosis assessments, it is evident that Mg^2+^ promotes apoptosis and inhibits the proliferation of cervical carcinoma cells. Our investigations reveal that high concentrations of 20 mM Mg^2+^ effectively inhibit proliferation and induces apoptosis of SiHa cells. As shown in Fig. 4, Our results showed that Mg^2+^ restrains the proliferation of SiHa cells by causing cell cycle arrest at the G0/G1 phase. Thus, Mg^2+^ not only elicits tumor cell death but also demonstrates benign effects on normal cells within appropriate concentration ranges. In vitro experiments have underscored the pivotal role of p53 in tumor progression and apoptosis, yet the precise contribution of p53 to medical anti-tumor therapy remains elusive. Our research, as shown in Fig. 6, support that different concentrations of Mg^2+^ can participate in p53-induced tumor cell apoptosis, and the higher the concentration is, the stronger the inhibition is. We can conclude that Mg^2+^ play a major role to inhibit the growth of cervical cancer. To further verify the inhibitory effect of Mg^2+^ on cervical cancer, we conducted in vivo research, as shown in Fig. 7. In our study, subcutaneous tumors in nude mice were subjected to treatment with varying concentrations of Mg^2+^, while a blank control group was utilized to assess the specific impact of Mg^2+^. Figure 7H shows that 15 mM and 20 mM Mg^2+^ treatment groups significantly inhibited the growth of SiHa cells in vivo, and the tumor volume in the 20 mM Mg^2+^ group was smaller than that in the other control groups. Figure 7I results showed that compared with the control group, Mg^2+^ have a certain inhibitory effect on tumor. In addition, the higher the concentration of Mg^2+^, the more promoted the necrosis and reduction of tumor tissue, especially in the 15 mM Mg^2+^ and 20 mM Mg^2+^ groups of tumor tissue, which showed significant fibrous tissue proliferation and infiltration of inflammatory cells while tumor tissue decreasing. Studying the impact of various Mg^2+^ concentrations on cervical cancer cells revealed significant cytotoxicity, with higher concentrations demonstrating stronger inhibition of cancer cells. This suggests that Mg^2+^ may inhibit tumor growth by enhancing the immune response. The findings underscore the potential of Mg implants as a viable clinical approach for cervical cancer treatment, offering a solid theoretical foundation for future use of Mg-based metallic materials. Further exploration is needed to elucidate the precise mechanisms underlying Mg^2+^’s inhibitory effects on cervical cancer cells.

## Conclusion

Across both in vitro and in vivo experiments, we have observed a significant inhibitory effect of varying concentrations of Mg^2+^ on cervical cancer. The study provides evidence that Mg^2+^ effectively impedes the proliferation of tumor cells by inducing cell cycle arrest, particularly in the G0/G1 phase. Moreover, exposure of SiHa cells to Mg^2+^ leads to suppressed tumor cell proliferation accompanied by apoptosis. This effect was further validated by in vivo experiments, wherein the 20 mM Mg^2+^ group exhibited the smallest tumor volume. In summary, these findings suggest that the degradation products of Mg, particularly the released 20 mM Mg^2+^, exert a potent inhibitory influence on the biological characteristics of cervical cancer. This study establishes a theoretical basis for the potential utilization of Mg-based biomaterials in future cervical cancer treatments. However, further research and exploration are needed to elucidate the specific mechanisms by which Mg-based metal materials exert anti-tumor effects.

## Methods

### Material preparation and sterilization

To prepare the magnesium chloride solution, dissolve anhydrous MgCl_2_ (99.99%, Sigma Aldrich, USA) in deionized water. Filter the solution using a 0.22 μm filter from Corning (USA), and subsequently dilute it with cell culture medium to create concentrations of 5 mM, 10 mM, 15 mM, and 20 mM Mg^2+^. The experiment was divided into A: complete A-MEM (Hyclone, USA) medium control groupn(NC), B: 5 mM Mg^2+^ treatment group, C: 10 mM Mg^2+^treatment group, and D: 15 mM Mg^2+^group. E: 20 mM Mg^2+^treatment group.

All the methods are reported in accordance with ARRIVE guidelines and were carried out in accordance with other relevant guidelines and regulations.

## In vitro experiments

### Cell culture

The human cervical cancer cell lines SiHa, were sourced from the cell repository of the Chinese Academy of Sciences (CBCAS, Shanghai, China). These cells were cultured in McCoy’s 5A medium (Thermo Scientific, MA, USA), supplemented with 10% fetal bovine serum (FBS), and 1% penicillin and streptomycin (Thermo Scientific), while being maintained at a temperature of 37 °C with 5% CO_2_.

### Cell proliferation test

To take SiHa cells with logarithmic growth phase, perform cell counting, adjust cell concentration, and follow 1 × 10^4^/well into a 96 well plate. Incubated in a constant temperature incubator at 37 °C with 5% CO_2_ for 24, 48, and 72 h. Remove the culture medium and clean each well three times with 100 μl PBS. Add culture medium containing 100 μl CCK-8 (Solarbio, China) to each wells, and incubate for 2 h in a 37 °C constant temperature incubator. Detect the absorbance value at 450 nm using an enzyme-linked immunosorbent assay.

### Staining of live/dead cell detection

Conduct the LIVE/DEAD cell assay following the guidelines provided by the manufacturer (LIVE/DEAD staining kit, Biotium, California, United States). To put it simply, seed the cells into a 48-well culture plate and allow them to grow overnight in McCoy’s 5A medium. Subsequently, for the next 48 h, substitute the medium with either (a) pH-adjusted or (b) Mg^2+^-enriched culture medium. Carefully rinse the harvested cells with PBS and then treat them with 5 μl of Calcein AM/4 and 20 μl of EthD-II in PBS at room temperature for a duration of 30 min. Following this, replace the solution with fresh culture medium and capture images of the cells using an inverted fluorescence microscope.

### ROS detection

To detect intracellular ROS, the reagent kit (Mlbio, China) was used. Briefly, trypsin-hydrolyzed cells were set to a density of 3 × 10^5^/ml. The supernatant was removed, and the remaining cells were dissolved in 50 μl test solution. Next, 100 μl of enzyme solution was added. All test samples were incubated at 37 °C for 60 min. After washing the microtitration plate four times, transfer 50 μl of Matrix A and 50 μl of Substrate B into each well using a pipette. Gently mix the microplates and incubate them at 37 °C for 15 min. Next, replace the spent culture medium with fresh medium and capture cell images using an inverted fluorescence microscope.

### Cell cycle analysis

The cells were nurtured in McCoy medium, subsequently cleansed with cold PBS, and left to incubate overnight in 70% cold ethanol at 20 °C. Following another cold PBS wash, the cells were prepared for the next steps. They were placed in a buffer chamber and subjected to PI/RNase staining, where the process unfolded in a darkened environment at the appropriate temperature for 30 min. The subsequent step involved the use of flow cytometry to observe and analyze the cell cycle distribution.

### Cell apoptosis analysis

Following a 48-h incubation period in media featuring varying Mg^2+^ (5, 10, 15, 20 mM), SiHa cells were collected for apoptosis assessment. Subsequently, the cells were divided, with each group consisting of cells resuspended at a density of 10^6^ cells/ml. A 100 µl cell suspension was transferred to 1.5 ml test tubes and subjected to staining using 5 µl of FITC annexin V and 10 µl of propidium iodide solution. The cells were gently vortexed and allowed to incubate at room temperature for 15 min. 400 µl of annexin V binding buffer was introduced to test tube, thoroughly mixed, and the resulting mixture was subjected to analysis through flow cytometry (CytoFLEX S, Beckman Coulter, CA, USA).

### Western blotting

Place SiHa cells in a medium containing 10% FBS α-Cultivate cells in MEM complete medium for 7 days using IP (Beyotime, China) lysis culture. Then, protein concentration was measured using BCA protein quantification (Beyotime, China). The protein sample was heated and dried at 98 °C for 5 min to denature the protein, and loaded onto the SDS-PAGE gel. Then transfer the protein to a suitable PVDF membrane. Incubate the membrane in a closed solution (5% BSA) at room temperature for two hours, and then wash with TBST washing solution. After washing, the p53 antibody was added and the membranes were incubated overnight at 4 °C. subsequently, the membranes were incubated with a secondary antibody for 90 min at room temperature. after washing with TBST, the reaction was performed with a chemiluminescent reagent and exposure was performed. The Western blot images were semi-quantitatively analyzed by using Image J.

### Statistical analysis

Analysis was subjected to each group with a minimum of three samples. All collected data underwent statistical assessment utilizing one-way ANOVA. Statistical comparisons were made to discern significant distinctions between the various sample groups. Quantitative data is expressed as the mean ± standard deviation for each respective group. A *p*-value of less than 0.05 was deemed indicative of statistical significance.

## In vivo experiments

After one week of adaptive animal feeding, the nude mouse were anesthetized by intraperitoneal injection of 60mg/kg pentobarbital sodium. After anesthesia, fix each animal onthe operating table, perform routine skin preparation, and disinfect it. SiHa cell lines were subcutaneously inoculated on the right side of the tested nude mice, with each mouse receiving 1 × 10^7^cells. When the tumor volume is approximately 70mm^3^-100mm^3^, a random stratified grouping method is used to start grouping based on the tumor volume and animal weight. Randomly divide into 5 experimental groups (control group, 5 mM Mg^2+^ treatment group, 10 mM Mg^2+^ treatment group, 15 mM Mg^2+^group, 20 mM Mg^2+^treatment group). Animal Strain (Balb/c nude mouse) Manufacturer (Shanghai Slake Experimental Animal). Gender(3 males and 3 females in each group). Age: 6–8 w. Quantity (20–25 g). CertificateNo: SCXK (Shanghai)2022-0004, No.20222004018368. The control group was injected with physiological saline,while the other groups were injected with 5 mM Mg^2+^, 10 mM Mg^2+^, 15 mM Mg^2+^, 20 mM Mg^2+^,with a dosage volume of 200 ul per group. The mice were euthanized (intraperitoneal injection of pentobarbital sodium 100 mg/kg) and the tumor was removed from the miceat 7 days. In addition, remove the mouse tumor and measure its volume, weight, and takephotosof its morphology, fix with formalin, embed in paraffin, and stain with hematoxylinand eosin (H&E). Measure the tumor size and weigh the mouse body weight on days 0, 3, 5, and7, and take photos of the mice. The calculation method for tumor volume is as follows: tumor volume = (L × W^2^)/2, where L is the length of the tumor and W is the width of the tumor. The animal study was reviewed and approved by Experimental Animal Ethics Committee of Xinjiang Medical University, ethical approval number(IACUC-2022 0725-24).

## Supplementary Information

Supplementary Information 1.

Supplementary Information 2.

Supplementary Information 3.

## Data Availability

Data will be made available on request. Mendeley Data, V1, https://doi.org/10.17632/jx9ndzk4kn.1.
