# Supplementary figures and images for: Biodegradable magnesium based metal materials inhibit the growth of cervical cancer cells
Source: Sci Rep. 2024 Sep 2;14:19155. doi: 10.1038/s41598-024-63174-w (PMC11369255; doi:10.1038/s41598-024-63174-w)

## WB-GAPDH(N1)

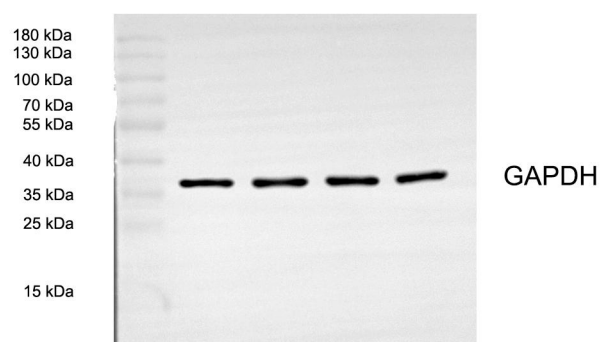

## WB-P53(N1)

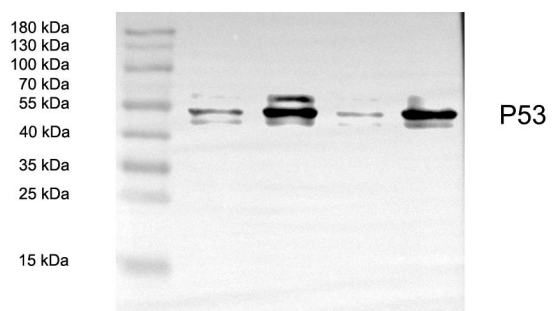

## WB-GAPDH(N2)

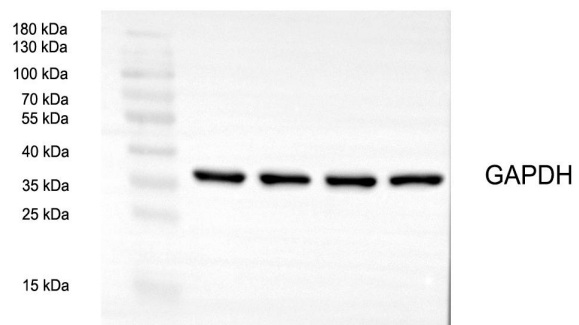

## WB-P53(N2)

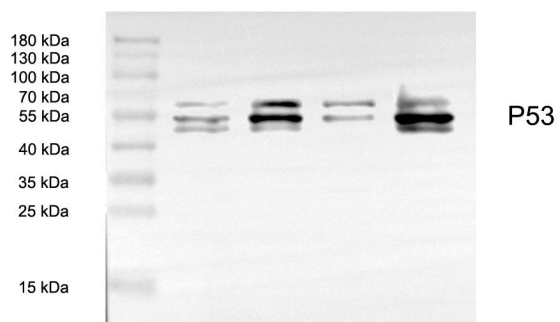

## WB-GAPDH(N3)

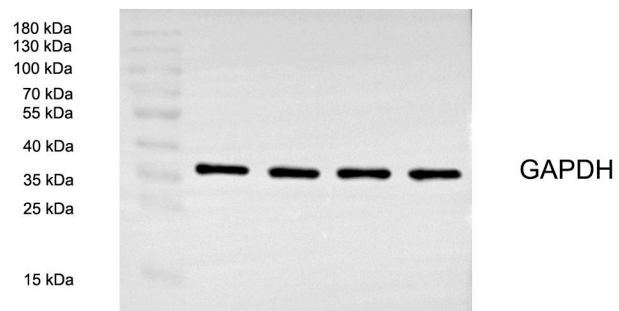

## WB-P53(N3)

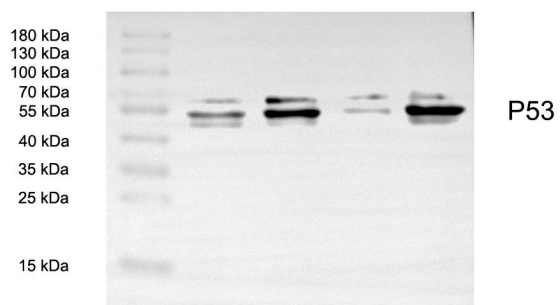

Supplement: Supplementary file 2 — Supplementary Information 2. [file 41598_2024_63174_MOESM2_ESM.pdf]
